# Mathematical Modelling of Alternative Pathway of Complement System

**DOI:** 10.1007/s11538-020-00708-z

**Published:** 2020-02-15

**Authors:** Suruchi Bakshi, Fraser Cunningham, Eva-Maria Nichols, Marta Biedzka-Sarek, Jessica Neisen, Sebastien Petit-Frere, Christina Bessant, Loveleena Bansal, Lambertus A. Peletier, Stefano Zamuner, Piet H. van der Graaf

**Affiliations:** 1 Division of Systems Biomedicine and Pharmacology, LACDR, Leiden University, P.O. Box 9502, 2300 RA Leiden, The Netherlands; 2 Certara QSP, 4818 SJ Breda, The Netherlands; 3 Cytokine, Chemokine and Complement DPU, Immunoinflammation TA Unit, GSK, Stevenage, UK; 4 Computational and Modelling Sciences, Platform Technology Sciences, GSK, Collegeville, Pennsylvania USA; 5 Mathematical Institute, Leiden University, P.O. Box 9512, 2300 RA Leiden, The Netherlands; 6 Clinical Pharmacology, Modelling and Simulation, GSK, Stevenage, UK; 7 Certara QSP, Canterbury, CT2 7FG UK

**Keywords:** Alternative pathway, Complement system, Immunology, C3 glomerulopathy

## Abstract

The complement system (CS) is an integral part of innate immunity and can be activated via three different pathways. The alternative pathway (AP) has a central role in the function of the CS. The AP of complement system is implicated in several human disease pathologies. In the absence of triggers, the AP exists in a time-invariant resting state (physiological steady state). It is capable of rapid, potent and transient activation response upon challenge with a trigger. Previous models of AP have focused on the activation response. In order to understand the molecular machinery necessary for AP activation and regulation of a physiological steady state, we built parsimonious AP models using experimentally supported kinetic parameters. The models further allowed us to test quantitative roles played by negative and positive regulators of the pathway in order to test hypotheses regarding their mechanisms of action, thus providing more insight into the complex regulation of AP.

**Electronic supplementary material:**

The online version of this article (10.1007/s11538-020-00708-z) contains supplementary material, which is available to authorized users.

## Introduction

**Fig. 1:**
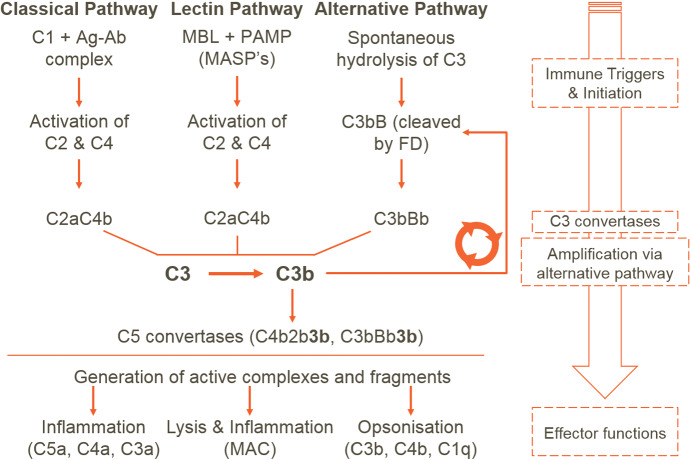
Schematic representation of the CS response. The classical pathway is triggered by binding of C1 to an Ag–Ab complex, whereas the lectin pathway is triggered by binding of MBLs to specific carbohydrate structures on pathogens (PAMP). Initiation of both these pathways leads to the hydrolysis of C2 and C4 generating C2a and C4b which form the classical and lectin pathway C3 convertase C4bC2a. Uniquely, the AP is triggered by the spontaneous hydrolysis of C3, a zymogen present at high concentrations in serum. The resulting C3(H$_2$O) binds factor B (FB), and the resulting pro-convertase complex is activated by factor D (FD) to yield the C3 convertase C3bBb. These initial C3 convertases further activate AP, which then leads to amplification by the AP. The terminal pathway is then activated, which leads to cleavage of C5 and MAC formation. The end-effects of CS activation are inflammation (via AP and terminal pathway), pathogen and cell lysis (by MACs) and opsonization (i.e. marking of pathogen surfaces for clearance, by-products of classical pathway, lectin pathway and AP activation) (Color figure online)

The complement system (CS) is a part of the innate immune system and bridges innate and adaptive immunity. CS provides first-line defence against microbes. It is also required for clearance of apoptotic cells and immune complexes. Several soluble and cell-surface proteins are involved in function and regulation of the CS. The CS is a proteolytic cascade and can be activated via classical, lectin and alternative pathways (AP). The pathways differ in the initial triggers; the classical pathway is triggered by interaction of complement component 1 (C1) with antigen–antibody complexes (Ag–Ab), whereas the lectin pathway LP is triggered by interaction of Mannose-binding lectins (MBLs) to specific carbohydrate structures on pathogens (PAMPs). Both classical and lectin pathway activation results in cleavage of C2 and C4. The AP, on the other hand, is constitutively active at low levels due to spontaneous hydrolysis of its precursor protein, namely C3. Following activation, all three pathways converge at the C3 level, where the amplification loop of the AP (Fig. 1) provides rapid amplification and downstream transmission of signals (Melis et al. 2015). All three pathways lead to activation of the “terminal pathway.” This is a series of reactions that generate the potent anaphylatoxin, C5a, as well as lethal molecular complexes (membrane attack complexes (MACs)) on cell surfaces (Melis et al. 2015).

The CS is believed to show a dichotomous response. In health (i.e. in the absence of triggers), it exists in a resting state as judged by the levels of precursor proteins (Alper and Rosen 1967, 1984; Scholl et al. 2008). The resting state is maintained through a range of fluid-phase and cell-surface regulatory proteins, including Factor H (FH), Factor I (FI) and Complement receptor 1 (CR1). On challenge with a trigger and on unprotected surfaces (e.g. through loss of regulator proteins), however, the CS can respond quickly and vigorously thereby resulting in generation of complement cleavage fragments and depletion of complement components (Pangburn et al. 1981; Fredrikson et al. 1993; Korotaevskiy et al. 2009). The CS has been implicated in several autoimmune and inflammatory diseases such as systemic lupus erythematous, paroxysmal nocturnal hemoglobinuria and ischaemia/reperfusion (I/R) injury (Chen et al. 2010; Kirschfink and Mollnes 2003; Melis et al. 2015). Dysregulation of AP, in particular, has been implicated in autoimmune and inflammatory disorders such as atypical haemolytic uraemic syndrome (aHUS), C3 glomerulopathy and age-related macular degeneration (AMD) but also conditions such as asthma and I/R injury (Thurman and Holers 2006; Zipfel et al. 2006; Anderson et al. 2010; Morgan and Harris 2015). It has been shown that the AP is responsible for up to 80% of complement response even when the activation is through the classical pathway (Harboe et al. 2004). The central role played by the AP in transmission as well as amplification of activation signals makes it an attractive candidate for therapeutic intervention (Holers and Thurman 2004).

In this work, we have focused on modelling the AP. The classical pathway was the first of the complement pathways to be mathematically modelled (Havsteen and Varón 1990; Hirayama et al. 1996). The AP was modelled in 2009 by Korotaevskiy and co-workers. The authors modelled the full nonlinear dynamics of the classical pathway as well as the AP (Korotaevskiy et al. 2009). The 2009 model, however, only focused on the acute activation response of the CS and did not explore the steady-state behaviour. The modelling effort was greatly enhanced by Zewde and co-workers through addition of cell-surface reactions as well as a detailed description of negative regulators of the CS (Zewde et al. 2016; Zewde and Morikis 2018). The most comprehensive of these models contains 290 variables and over 140 parameter values.

Except for the model presented in Sagar et al. (2017) and Zewde and Morikis (2018), all other models to date focus on acute activation of AP. This acute activation with concurrent depletion of complement components is not compatible with timescales of chronic conditions. The role of the complement in chronic autoimmune diseases, together with clinically observed complement component levels in these diseases, suggests that one may need to consider a (higher than basal) steady-state response in such cases, which may resemble “chronic low-level activation.” To this end, the work from Zewde and Morikis (2018) considers the effect of reduced FH—a negative regulator of AP activation, on the steady-state response.

Synthesis and degradation of precursors may be important in studying such a response, but has not been considered in any of the previous models. Furthermore, the larger models involve higher parameter uncertainty due to the lack of kinetic data around several modelled processes (see, for example, Zewde et al. 2016; Zewde and Morikis 2018). This has implications for the conclusions reached in these studies. A parsimonious model with high certainty parameter values is likely to provide more insight into the interplay of the pathway’s components. A reduced order model by Sagar et al., though parsimonious, does not consider the dynamics of factor B (FB) a key protein in AP, which gets consumed during AP activation (Sagar et al. 2017).

In the present study, we constructed two parsimonious models of AP with the aim to understand the steady-state response of the pathway under negative regulation and the effects of positive regulation. We began by constructing a minimal model required to produce a physiological steady state. We then added the only known positive regulator of the pathway, properdin, to determine the effect of positive regulation on the steady state. Finally, we used these models to understand quantitative roles played by the regulators in the pathway, which allowed us to verify hypotheses around the mechanisms of regulation.

## Model Development

**Fig. 2:**
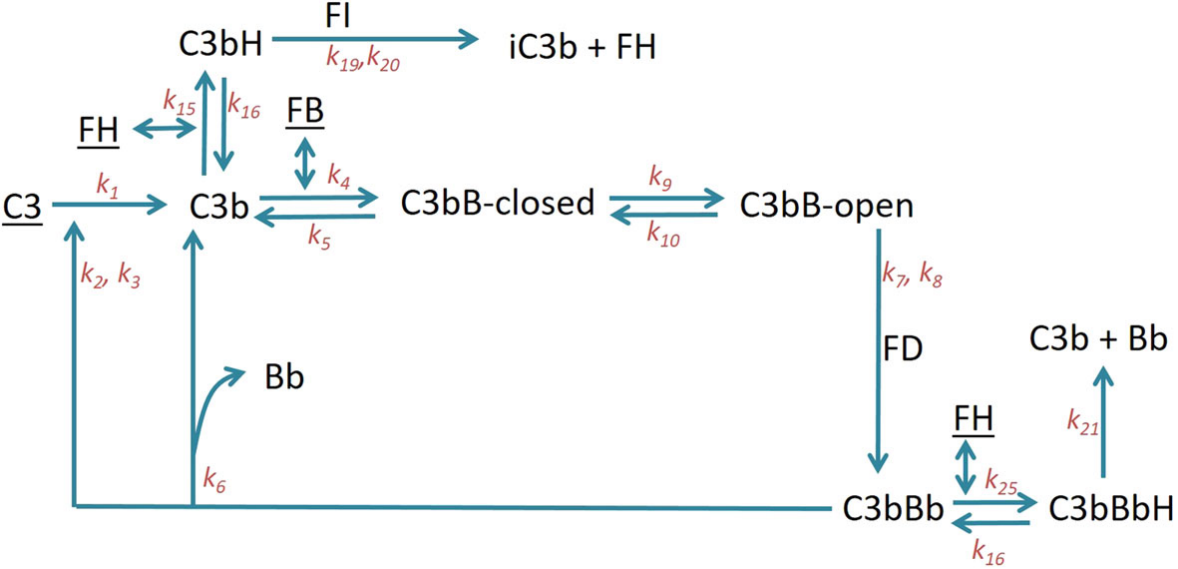
Schematic representation of the reactions in the minimal model of AP. Underlined components are subject to synthesis and degradation reactions, which are included in this model but excluded from the diagram for clarity. Reaction rate constants are indicated on the respective reaction arrows (Color figure online)

**Fig. 3:**
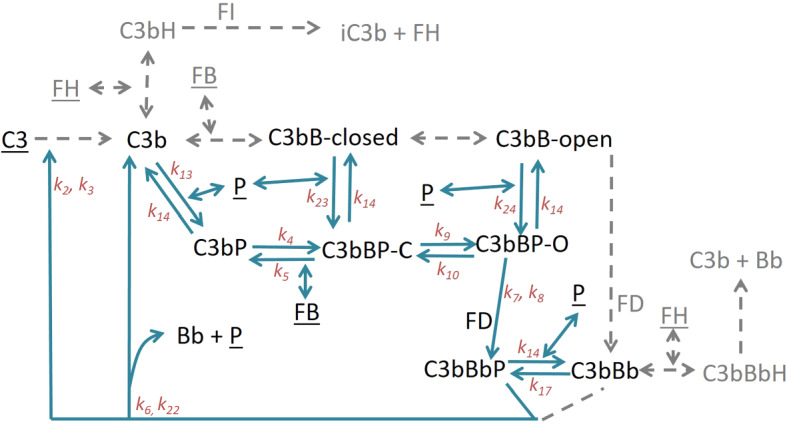
Schematic representation of the reactions in the properdin model. Properdin model is an expansion of the minimal model. The components and reactions from the minimal model, which do not bind properdin, are greyed out and simplified for better clarity. However, these reactions are modelled exactly as in the minimal model. Underlined components are subject to synthesis and degradation reactions, which are included in this model but excluded from the diagram for clarity. Reaction rate constants are indicated on the respective reaction arrows (Color figure online)

We present two AP models—(1) the minimal model and (2) the properdin model. The minimal model uses minimal machinery required to produce a physiological steady state and includes negative regulation. The properdin model includes the positive regulation by properdin in addition to the negative regulation in the minimal model. Figures 2 and 3 schematically show the reactions in the minimal and properdin models, respectively.

### Minimal Model

The AP rapidly amplifies signals from all three complement pathways. This is a result of the amplification loop in which C3b, an activation product of classical, lectin and alternative pathways, feeds into the formation of additional AP C3 convertase, C3bBb (see below), which in turn cleaves more C3 molecules and leads to further AP activation. AP reactions begin with spontaneous hydrolysis of C3 into C3($\hbox {H}_{{2}}$O). C3($\hbox {H}_{{2}}$O) is chemically different but functionally similar to C3b generated from enzymatic cleavage of C3. In this work, C3($\hbox {H}_{{2}}$O) and C3b are both represented as the same species C3b. C3b binds Factor B (FB) to form the C3bB pro-convertase. The pro-convertase can exist in two structural conformations, closed and open (Torreira et al. 2009). This conformational change has never been modelled despite well-described kinetic parameters being available (Table 1). The open form alone can bind the protease Factor D (FD) (Forneris et al. 2010; Hourcade and Mitchell 2011), which cleaves the FB part of the complex into Ba and Bb. Bb remains bound to C3b, thus forming C3bBb—the AP C3 convertase. Ba is released as a by-product. C3bBb, itself a protease, can cleave C3 into C3b and C3a, thus forming the feedback loop. C3a is an anaphylatoxin (Rooijakkers et al. 2009), but does not participate further in AP activation, and hence is treated as a by-product in the model. AP reactions mentioned above not only occur on pathogenic surfaces, but also in serum (fluid phase). In the present work, we focus on the latter, i.e. on the fluid-phase reactions.

In the absence of immune triggers, the AP is expected to be in homoeostasis, largely due to the stabilizing effect of negative regulation. FH and FI are the most abundant fluid-phase negative regulators of the AP. Additional regulators such as FH-like protein 1 (FHL-1) have been known, but due to the low concentration of FHL-1 in humans compared to FH, it is believed not to contribute substantially to systemic regulation of AP (Dopler et al. 2019). For this reason, we focus on FH. FH binds C3b to form the complex C3bH, which leads to the inactivation of C3b to iC3b by the action of serine protease FI. FI is unable to inactivate C3b in the absence of FH (Whaley and Ruddy 1976; Pangburn and Mueller-Eberhard 1983). FH additionally works by accelerating the decay of the C3 convertase C3bBb by displacing Bb (Harder et al. 2016).

Additionally, the synthesis and degradation reactions of pathway precursors such as C3, FB and FH are also included. Model equations are presented in “Appendix” (Eq. A.1).

A variant of this model the “truncated minimal model” was generated to simulate FH depletion or dysfunction by setting the FH synthesis rate and initial FH concentration to zero. This addition to the model is useful to simulate human disease where FH becomes dysfunctional resulting in unregulated activation of the AP. We discuss this variant further in Sect. 3.1.1.

Other cell-surface-based negative regulators of AP exist, namely complement receptor 1 (CR1) and decay-accelerating factor (DAF) (Sarma and Ward 2011), but have been omitted from this model, the implications of which are discussed in the results section.

### Properdin Model

Properdin (P) is the only known positive regulator of the AP and has previously been validated as a potential target for therapeutic intervention (Chen et al. 2018). It is believed to act by prolonging the half-life of C3 convertase C3bBb (Fearon 1975; Hourcade 2006). Properdin exists as a mixture of di-, tri- and tetra-mers (Sun et al. 2004) and has been shown to interact with the AP intermediates C3b, C3bB and C3bBb (Hourcade 2006). The properdin multimers can bind to more than one C3b molecule, thereby increasing the local concentration of C3b. However, since we are only concerned with fluid-phase reactions we restrict to monovalent binding between properdin and C3b. We include association/dissociation of monomeric properdin with/from C3b, C3bB (both closed and open forms) and C3bBb (Fig. 3). Model equations are presented in “Appendix” (Eq. C.1).

### Model Analysis and Simulations

The steady states of the models were determined through mathematical steady-state analysis (minimal model) or by simulations. Simulations were performed using a stiff differential equation solver ode15s from MATLAB R2015b. MATLAB code corresponding to various figures is available as supplementary material. Parameter values used for simulations are presented in Table 1. The parameters that were not directly available in literature, but had to be calculated, are indicated with a description of methods used to calculate them.

**Table 1:** Parameters used in various models together with their meaning, units, values and source

P	Value	Unit	Meaning	Reference
$k_{1}$	0.0001	$\hbox {min}^{-1}$	Tickover	Pangburn et al. (1981)
$k_{2}$	107	$\hbox {min}^{-1}$	MM rate for C3bBb-mediated lysis of C3	Pangburn and Muller-Eberhardt (1986)
$k_{3}$	5.86	$\upmu \hbox {M}$	$K_{M}$ for C3bBb-mediated lysis of C3	Pangburn and Muller-Eberhardt (1986)
$k_{4}$	0.816	$\upmu \hbox {M}^{-1}\,\hbox {min}^{-1}$	Binding rate of C3b and FB	Harris et al. (2005), Hourcade and Mitchell (2011) and Rooijakkers et al. (2009)
$k_{5}$	6.9	$\hbox {min}^{-1}$	Dissociation rate of closed C3bB	Harris et al. (2005), Hourcade and Mitchell (2011) and Rooijakkers et al. (2009)
$k_{6}$	0.46	$\hbox {min}^{-1}$	Dissociation rate of C3bBb	Pangburn and Muller-Eberhardt (1986)
$k_{7}$	130	$\hbox {min}^{-1}$	MM rate for FD-mediated lysis of open C3bB	Korotaevskiy et al. (2009)$^{\mathrm{b}}$
$k_{8}$	0.72	$\upmu \hbox {M}$	$K_{M}$ for FD-mediated lysis of open C3bB	Katschke et al. (2012)
$k_{9}$	0.33	$\hbox {min}^{-1}$	Conformational change rate for closed to open	Harris et al. (2005), Hourcade and Mitchell (2011) and Rooijakkers et al. (2009)
$k_{10}$	0.1068	$\hbox {min}^{-1}$	Conformational change rate for open to closed	Harris et al. (2005), Hourcade and Mitchell (2011) and Rooijakkers et al. (2009)
$k_{13}$	0.87	$\upmu \hbox {M}^{-1}\,\hbox {min}^{-1}$	Binding rate for C3b and properdin	DiScipio (1981) and Hourcade (2006)$^{\mathrm{a}}$
$k_{14}$	0.03	$\hbox {min}^{-1}$	Dissociation rate for C3bP (or C3bBP)	Hourcade (2006)
$k_{15}$	312	$\upmu \hbox {M}^{-1}\,\hbox {min}^{-1}$	Binding rate for C3b and FH	Pangburn and Mueller-Eberhard (1983)$^{\mathrm{c}}$
$k_{16}$	195	$\hbox {min}^{-1}$	Dissociation rate for C3b and FH	Pangburn and Mueller-Eberhard (1983)
$k_{17}$	$k_{13}$	$\upmu \hbox {M}^{-1}\,\hbox {min}^{-1}$	Binding rate for C3bBb and properdin	DiScipio (1981) and Hourcade (2006)
$k_{18}$	0.028	$\hbox {min}^{-1}$	Dissociation rate for C3bBbP	Hourcade (2006)
$k_{19}$	78	$\hbox {min}^{-1}$	MM rate for FI-mediated inactivation of C3bH	Pangburn and Mueller-Eberhard (1983)
$k_{20}$	0.25	$\upmu \hbox {M}$	$K_{M}$ for FI-mediated inactivation C3bH	Pangburn and Mueller-Eberhard (1983)
$k_{21}$	**0.832**	$\hbox {min}^{-1}$	Dissociation rate for C3bBbH into C3b, Bb and FH	Harder et al. (2016)$^{\mathrm{d}}$
$k_{22}$	0.023	$\hbox {min}^{-1}$	Dissociation rate for C3bBbP into C3b, Bb and properdin	Hourcade (2006)
$k_{23}$	0.87	$\upmu \hbox {M}^{-1}\,\hbox {min}^{-1}$	Binding rate for closed C3bB and properdin assumed equal to $k_{13}$	DiScipio (1981) and Hourcade (2006)
$k_{24}$	0.87	$\upmu \hbox {M}^{-1}\,\hbox {min}^{-1}$	Binding rate for open C3bB and properdin assumed equal to $k_{13}$	DiScipio (1981) and Hourcade (2006)
$k_{25}$	312	$\upmu \hbox {M}^{-1}\,\hbox {min}^{-1}$	Binding rate for C3bBb and FH assumed equal to $k_{15}$	Pangburn and Mueller-Eberhard (1983)$^{\mathrm{c}}$
$k_{s1}$	0.00316	$\upmu \hbox {M}\,\hbox {min}^{-1}$	Synthesis rate of C3	Alper and Rosen (1984)
$d_{1}$	0.000392	$\hbox {min}^{-1}$	Degradation rate of C3	Alper and Rosen (1984)
$k_{s2}$	0.000798	$\upmu \hbox {M}\,\hbox {min}^{-1}$	Synthesis rate of FB	Alper and Rosen (1984)
$d_{2}$	0.000333	$\hbox {min}^{-1}$	Degradation rate of FB	Alper and Rosen (1984)
$k_{s3}$	0.00067	$\upmu \hbox {M}\,\hbox {min}^{-1}$	Synthesis rate of FH	Charlesworth et al. (1979) and Dopler et al. (2019)$^{\mathrm{e}}$
$d_{3}$	0.00022	$\hbox {min}^{-1}$	Degradation rate of FH	Charlesworth et al. (1979) and Alper and Rosen (1984)
$k_{s4}$	0.00007	$\upmu \hbox {M}\,\hbox {min}^{-1}$	Synthesis rate of properdin	Ziegler et al. (1975)
$d_{4}$	0.000016	$\hbox {min}^{-1}$	Degradation rate of properdin	Ziegler et al. (1975)
FD	0.08	$\upmu \hbox {M}$	Concentration of FD	Alper and Rosen (1984) and Scholl et al. (2008)
FI	0.4	$\upmu \hbox {M}$	Concentration of FI	Grumach et al. (2006)
C3	5.4–8.6 (6)	$\upmu \hbox {M}$	Baseline concentration range of C3	Korotaevskiy et al. (2009)
			(Initial condition used in the model)	
FB	2–2.3 (2)	$\upmu \hbox {M}$	Baseline concentration range of FB	Zhang et al. (2014)
			(Initial condition used in the model)	
FH	1.7–3.3 (3)	$\upmu \hbox {M}$	Baseline concentration range of FH	Dopler et al. (2019)
			(Initial condition used in the model)	
P	0.14–0.64 (0.3)	$\upmu \hbox {M}$	Baseline concentration range of properdin	Stover et al. (2015)
			(Initial condition used in the model)	

$^{\mathrm{a}}$ Value of $k_{13}$ is calculated using the reported $k_{14}$ value (Hourcade 2006) in combination with affinity of properdin to C3b (DiScipio 1981) assuming a standard 1:1 binding model.

$^{\mathrm{b}}$ Value of $k_{7}$ is available from computational estimations (Korotaevskiy et al. 2009).

$^{\mathrm{c}}$ The minimum value of $k_{15}$ was estimated by Pangburn and Mueller-Eberhard (1983), which we have used in the model.

$^{\mathrm{d}}$ Value of $k_{21}$ was estimated using graphical data available in Harder et al. (2016) under the assumption of exponential decay.

$^{\mathrm{e}}$ Value of synthesis rate of FH was calculated using its reported degradation rate $d_3$ and serum concentration in Dopler et al. (2019)

### Hypothesis Testing

FH negatively regulates the AP by interacting with both C3b and C3bBb. Similarly, properdin positively regulates the AP by forming interactions with C3b, C3bB and C3bBb. However, it is not known which of these interactions contribute most to the regulation by either regulator. The developed models were used to dissect the quantitative roles of the regulators, by constructing *in silico* mutants of the regulators with limited interaction potential.

Three modified minimal model variants were constructed. In the first, FH is unable to bind either C3b or C3bBb ($k_{15} = k_{25}=0$). This variant is functionally equivalent to the truncated minimal model. In the second, FH is unable to bind C3b ($k_{15}=0$) and in the third, it is unable to bind C3bBb ($k_{25}=0$). The model outputs were compared against the minimal model.

To test the quantitative role played by various properdin interactions, a series of different model variants were constructed. Firstly, the minimal model was altered by letting the half-life of C3bBb be equal to the longer-lived properdin bound form C3bBbP. Thus, we set $k_{6} = k_{22}$. This was done to simulate the half-life prolonging effect of properdin. No properdin is present in this model. Further, properdin model was modified to selectively include properdin binding to either C3b or C3bB or C3bBb, at a time. This was done by setting different binding rates to zero as shown in Table 2. The aim was to capture binding interactions which are most influential in making a minimal model output resemble the properdin model output.

**Table 2:** Table comparing the series of model variants constructed to dissect the most important contributors to the positive regulation by P

Name	Base model	Properdin included?	Parametric change
MM	MM	No	–
MM	MM	No	$k_{6} = k_{22} = 0.023$ min$^{-1}$
+ long lived C3bBb			
Properdin binds C3b	PM	Yes	$k_{17} = k_{23} = k_{24} = 0$
Properdin binds C3bB	PM	Yes	$k_{13} = k_{17} = 0$
Properdin binds C3bBb	PM	Yes	$k_{13} = k_{23} = k_{24} = 0$
Properdin model	PM	Yes	–

MM and PM refer to minimal model and properdin model, respectively. Parametric change refers to the change in the model variant with respect to the base model

## Results and Discussion

We present the results of model simulations and validate them with experimental and modelling literature. We further use the models to test various hypotheses quantitatively. Relevant mathematical analyses are presented in the “Appendix.”

### Simulations, Analysis and Validation

#### Minimal Model

**Fig. 4:**
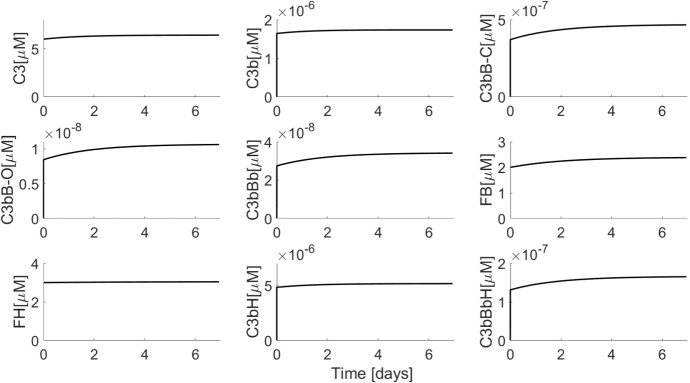
Simulation of minimal model (Eq. C.1) using parameter values from Table 1. Initial conditions used were C3 $= 6\,\upmu \hbox {M}$, FB = $2\,\upmu \hbox {M}$ and FH $= 3\,\upmu \hbox {M}$ and zero for all other variables

Figure 4 shows simulations of the minimal model. We began the simulations using physiologically realistic baseline values of the precursors C3, FB and FH. We chose zero as the initial concentrations of all the intermediates because they are generated from the precursors and because their physiological levels are not known. Choosing nonzero initial concentrations for intermediates only changes the initial trajectories of the solution and not the steady state reached. It can be seen that a steady state is indeed attained in which the concentrations of C3, FB and FH match the physiological concentrations observed experimentally (Table 3 in the “Appendix”) (Alper and Rosen 1984; Scholl et al. 2008). Simulated concentrations of intermediates cannot be validated experimentally as such data are not available. It is not possible to obtain a closed-form steady-state solution of the full minimal model. However, simulations revealed vast differences in concentration scales of variables. In particular, the AP intermediates concentrations were picomolar or lower, compared to micromolar concentrations of the precursors (C3, FB and FH). We used these differences in scales to obtain a reduced version of the minimal model, which was amenable for steady-state analysis (“Appendix A.1”). Evaluation of this analytical steady state using model parameters showed a close match with the simulated steady state of the full minimal model (Table 3 in the “Appendix”).

Further, we have nondimensionalized the model in order to gain insight into the relative impact of various pathway reactions (“Appendix A.2”). The nondimensionalization revealed that FH/FI-mediated reactions occur on a much faster time scale than many of the other reactions, whereas the synthesis and degradation reactions occur on a slow time scale. The dimensionless model may also be amenable to model reduction using singular perturbation analysis. However, such analysis is out of scope of the present work.

Attainment of healthy physiological C3 steady state in the presence of spontaneous C3 hydrolysis illustrates the strong negative regulation exerted by FH/FI. This is in agreement with previous modelling study (Zewde and Morikis 2018), even though the previous model does not include synthesis and degradation reactions. The extremely low intermediates concentrations indicate effective control of AP activation. An additional distinguishing feature of our model is the explicit inclusion of closed and open forms of C3bB. Previous models have considered only one form of C3bB (which was the closed form, based on parameter values chosen) (Zewde et al. 2016; Zewde and Morikis 2018). This implies an underlying assumption that closed to open C3bB conversion is fast, which is in contrast to kinetic literature. Inclusion of the two forms does result in attainment of a quantitatively different steady-state level, particularly of C3bBb and C3, as compared to the inclusion of just one form (not shown). Therefore, we believe that our model provides a more kinetically accurate description of the kinetics of C3bB.

Quantitative or functional depletion of FH is associated with some forms of C3 glomerulopathy and a common variant of FH (Y402H) increases the risk of developing AMD by approximately 7-fold (Hageman et al. 2005). This variant of FH has reduced binding to certain glycosaminoglycan which appear to be particularly important for protection of the retinal epithelium. In order to simulate FH depletion, we constructed a variant of the minimal model (called truncated minimal model) by setting FH synthesis rate and initial concentration to zero. This model shows strong activation response. After a delay, we observe severe C3 and FB depletion with corresponding rise in levels of all the intermediates, including C3bBb (results presented in “Appendix”). Cases of patients with severely depleted and clinically undetectable C3 levels have been noted (Pickering and Cook 2008). FB depletion is also observed concurrently to C3 depletion in SLE (Walport 2002) as well as in in vitro experiments with purified C3b, FB and FD (“Appendix D”, Fig. 10). A previous model by Sagar et al., has disregarded FB dynamics in their model (Sagar et al. 2017). This assumption is unlikely to have an effect on healthy steady-state model as the FB remains invariant in healthy state (Fig. 4). In case of a diseased model (such as the truncated model), however, simulating FB dynamics is crucial as the model predicts FB depletion, which is also observed clinically.

Typical cases of FH deficiency diseases show less severe C3 and FB depletion than that in truncated minimal model (Zhang et al. 2014). This suggests that an FH diseased state may lie somewhere between the two extremes (of minimal and truncated minimal model). Zewde and Morikis have used an approach of reducing FH concentration to study partial FH deficiency disease and found that an order of magnitude reduction in FH levels caused around 76% reduction in C3 levels. We are able to recreate this trend qualitatively by reducing FH synthesis rate and FH initial condition, although quantitative differences exist in the level of FH reduction necessary to achieve the same level of C3 reduction. The quantitative difference is most likely caused by different FH and C3b binding affinity used in their work (Zewde and Morikis 2018).

#### Properdin Model

Next, we questioned whether selective inclusion of negative regulation alone biases the model towards a steady-state response. We investigated whether adding properdin, accounting for all its interactions, to the minimal model would allow for the occurrence of a physiological steady state.

**Fig. 5:**
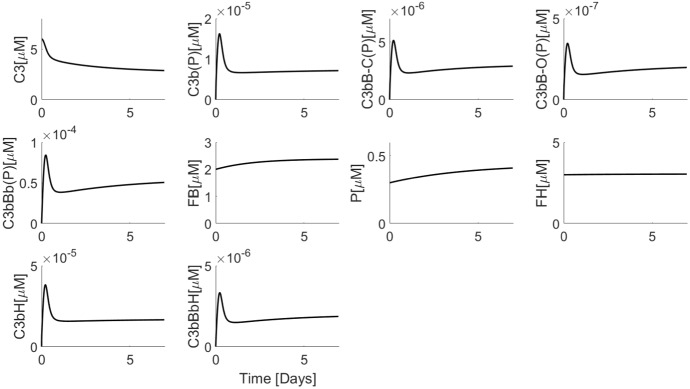
Simulation of properdin model (Eq. C.1) using parameter values from Table 1. Initial conditions used were C3 $= 6\,\upmu \hbox {M}$, FB $= 2\,\upmu \hbox {M}$, FH $= 3\,\upmu \hbox {M}$ and P $= 0.3\,\upmu \hbox {M}$ and zero for all other variables. Note that species which can exist in complex with properdin, such as C3b, are denoted with (P) in the end, to indicate that they refer to the total C3b concentration (i.e. C3b(P) = C3b + C3bP). The same notation is used for C3bB-C(P), C3bB-O(P) and C3bBb(P)

Simulation of the properdin model (Fig. 5) shows that it can predict the physiological steady-state levels for FB, FH and properdin (Alper and Rosen 1984; Scholl et al. 2008). However, C3 steady state is lower than the physiological level and lower than that predicted by the minimal model (Table 3 in the “Appendix”). Concomitantly, the total C3 convertase concentration (C3bBb(P)) (i.e. C3bBb + C3bBbP) is over 1000-fold higher than C3bBb levels in the minimal model. Thus, the reduction in C3 levels in comparison with the minimal model is due to the increased contribution from enzymatic cleavage of C3 by C3 convertase. Analytical steady-state solution of the properdin model is not possible due to the nonlinearity in the C3 equation (which is no longer small as in the minimal model).

Additional negative regulators of the pathway (e.g. CR1 and DAF) may rescue the reduction in C3 levels due to properdin. DAF works by accelerating decay of C3 convertases, thereby preventing further C3 cleavage. CR1 has both decay-accelerating activity as well as a co-factor activity in the FI-mediated inactivation of C3b. Both these are cell-surface-based regulators and are expressed on various circulating cells including erythrocytes (Noris and Remuzzi 2013). For this reason, they may augment FH function and play a role in systemic regulation of AP.

The simulation shows a non-monotonic approach to steady state, in contrast with the minimal model. The increased C3 convertase levels predicted indicate that the positive regulatory effect of properdin is captured by this model.

The above observations suggest that inclusion of properdin in the model might shift the balance towards an increased C3 convertase response, and away from the physiological steady state. It is possible that increased C3 convertase levels attained in the presence of properdin may lower the threshold of activation signals necessary to obtain an activation response. The minimal model is more suitable for simulating the physiological steady state due to the physiological C3 steady state predicted by it. A lack of information on physiological baseline levels of C3bBb(P) preclude the use of this marker to choose between the two models. Previous models have not included the interactions of properdin with C3b and C3bB in fluid phase or cell surface (Zewde et al. 2016; Zewde and Morikis 2018). As a result, simulations of these models cannot be compared to our results.

### Using Models for Hypotheses Testing

Regulators of AP can exert their effect at various points along the pathway. For example, properdin binds to C3b, C3bB as well as C3bBb. However, the quantitative roles of each of the interactions are based on speculation. We used hypothesis testing to determine the most influential interactions of the regulators in AP.

#### Mechanism of Action of FH

It is well understood that FH acts as a negative regulator by sequestering C3b, thereby making it unavailable for binding FB. FH is also known to bind C3bBb and increase its dissociation rate (Harder et al. 2016; Dopler et al. 2019). Previous models have also included this dual effect of FH on AP regulation (Zewde et al. 2016; Zewde and Morikis 2018); however, they have not compared the relative contribution of FH-mediated regulation at C3b vs C3bBb. We used our minimal model to test which of these two FH interactions have the greatest regulatory impact.

We performed this analysis by constructing three variants of the minimal model. In the first variant, FH was not allowed to bind either C3b or C3bBb (i.e. we set $k_{15} = k_{25} = 0$). In the second variant, FH was allowed to bind C3b alone, but not C3bBb ($k_{15} = 312~{\upmu \hbox {M}^{-1}\,\hbox {min}^{-1}}, k_{25} = 0$). In the third variant, FH was allowed to bind C3bBb alone and not C3b ($k_{15} = 0, k_{25} = 312~{\upmu \hbox {M}^{-1}\,\hbox {min}^{-1}}$).

**Fig. 6:**
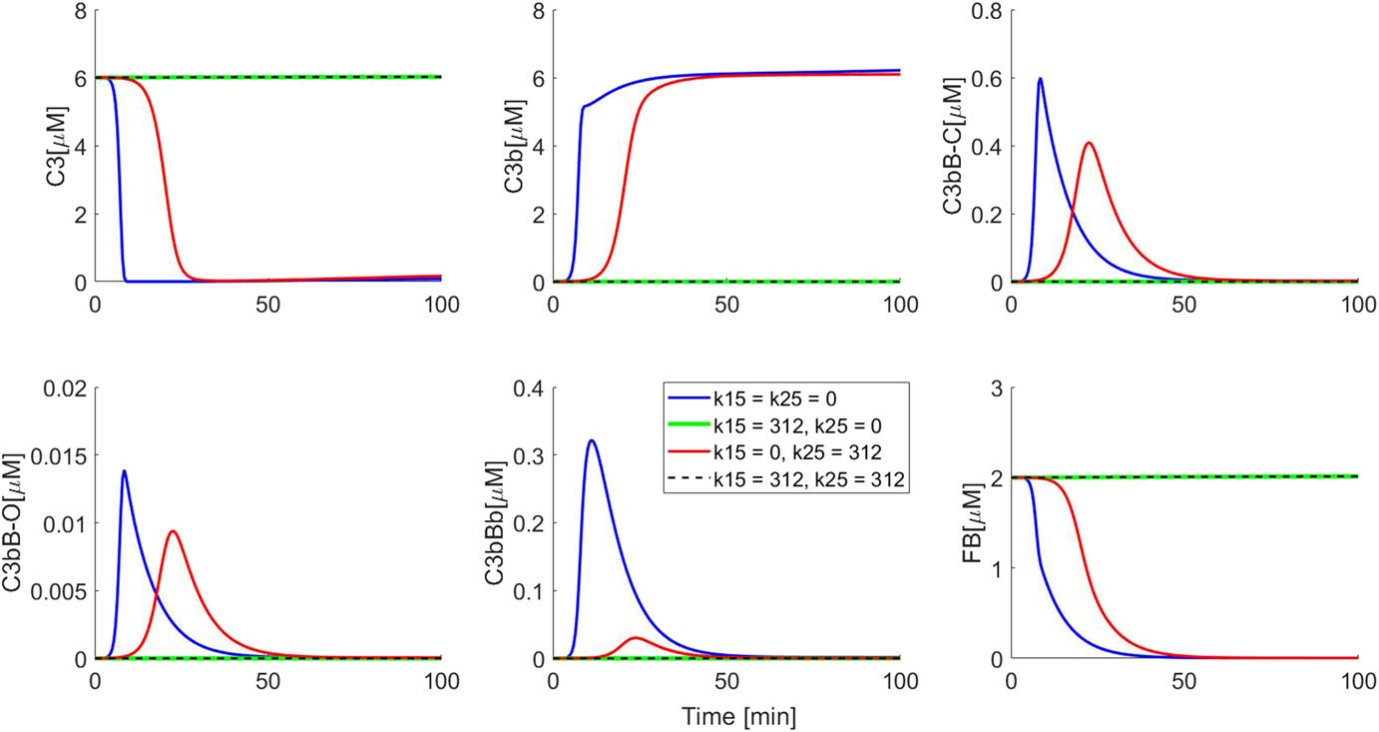
Simulations showing dissection of FH impact on the minimal model. Initial conditions used for all model variants were C3 $= 6\,\upmu \hbox {M}$, FB $= 2\,\upmu \hbox {M}$ and FH $= 3\,\upmu \hbox {M}$ and zero for all other variables. It can be seen that when FH binds C3bBb alone ($k_{15} = 0, k_{25} = 312~{\upmu \hbox {M}^{-1}\,\hbox {min}^{-1}}$), the resulting negative regulation is not strong enough to block activation as compared to when FH binds C3b alone ($k_{15} = 312~{\upmu \hbox {M}^{-1}\,\hbox {min}^{-1}}, k_{25} = 0$) or both C3b and C3bBb ($k_{15} = 312~{\upmu \hbox {M}^{-1}\,\hbox {min}^{-1}}, k_{25} = 312~{\upmu \hbox {M}^{-1}\,\hbox {min}^{-1}}$) (Color figure online)

The first variant is functionally identical to the truncated minimal model and generates the same strong activation response as the truncated model. We use this simulation output as a “negative control.” We found that allowing FH to bind C3bBb alone ($k_{15} = 0, k_{25} = 312~{\upmu \hbox {M}^{-1}\,\hbox {min}^{-1}}$) impacted the behaviour of the minimal model (Fig. 6). It can be seen that the resulting variant displays a qualitatively similar response to the truncated minimal model, in that it predicts complete C3 and FB depletion, albeit after a longer lag-phase than the truncated minimal model. The C3bBb levels were quantitatively closer to those observed in the truncated minimal model and significantly higher than in the minimal model.

When FH was allowed to bind C3b alone (second model variant, $k_{15} = 312~{\upmu \hbox {M}^{-1}\,\hbox {min}^{-1}}, k_{25} = 0$), on the other hand, the model behaved in a manner indistinguishable from the minimal model ($k_{15} = 312~{\upmu \hbox {M}^{-1}\,\hbox {min}^{-1}}, k_{25} = 312~{\upmu \hbox {M}^{-1}\,\hbox {min}^{-1}}$). This suggests that FH primarily regulates the AP by binding to and leading to the inactivation of C3b. The C3bBb-decay-accelerating role of FH appears to be less important for negative regulation. Nondimensionalization of the minimal model reveals that even though FH binding to C3b and C3bBb is equally fast, the clearance of C3bH (through FI-mediated inactivation) is faster than clearance of C3bBbH (via dissociation). This difference in scales may be responsible for the quantitative importance of C3b binding in negative regulation.

This observation is relevant as a number of pharmaceutical and academic groups are working on FH-like inhibitors for therapeutic intervention in complement-mediated disease (Hebecker et al. 2013; Schmidt et al. 2013; Nichols et al. 2015). Our data suggests development should focus on the compounds which enhance or mimic the FI co-factor effect.

#### Mechanism of Action of Properdin

The properdin model captures the positive regulatory effect of properdin. Properdin interacts with many components of AP including C3b, C3bB (both closed and open forms) and C3bBb (Hourcade 2006). However, it is not clear which of these interactions is more important. We simulated variants of the properdin model (as well as one variant of the minimal model) by setting various reaction rates to zero (see Table 2). The idea was to create in silico mutants of properdin with limited interaction potential to study the quantitative effect of properdin on AP. Notably, previous models of AP did not include interactions of properdin with C3b and C3bB in fluid-phase (Zewde et al. 2016; Zewde and Morikis 2018) and are, therefore, unsuitable to test this hypothesis.

**Fig. 7:**
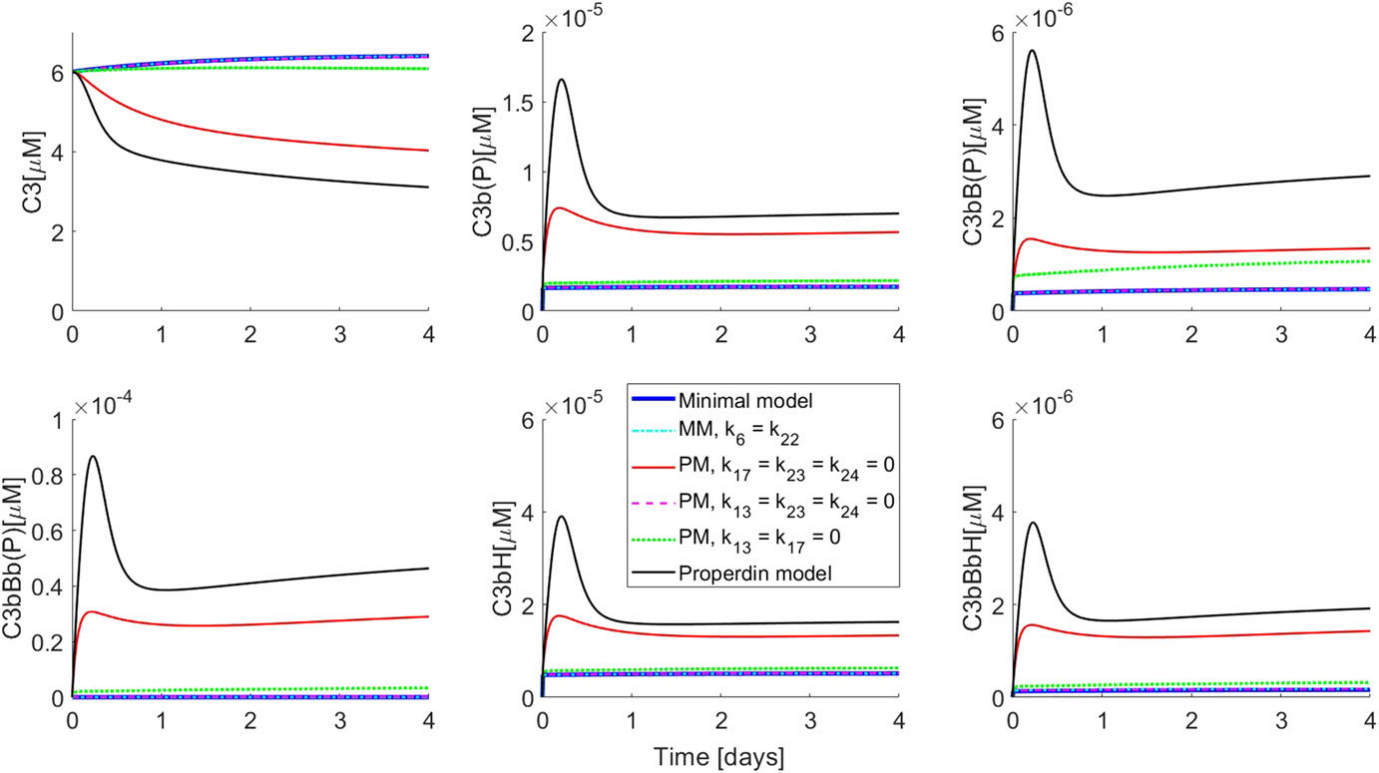
Simulations showing dissection of properdin impact on the minimal model. Initial conditions used for all model variants were C3 $= 6\,\upmu \hbox {M}$, FB $= 2\,\upmu \hbox {M}$, FH $=3\,\upmu \hbox {M}$ and P $= 0.3\,\upmu \hbox {M}$ and zero for all other variables. MM and PM denote minimal model and PM Properdin model, respectively. Note that species which can exist in complex with properdin, such as C3b, are denoted with (P) in the end, to indicate that they refer to the total C3b concentration (i.e. C3b(P) = C3b + C3bP). The same notation is used for C3bB-C(P), C3bB-O(P) and C3bBb(P) (Color figure online)

Figure 7 shows the results of simulations in comparison with the output from the minimal model (solid blue line; MM, in the figure legend) (without properdin and without increased C3bBb half-life) as well as with the properdin model (solid black line; PM in the figure legend). It is apparent that increasing the half-life of C3bBb in the minimal model (cyan dashed-dotted line; MM, $k_{6}=k_{22}$ in the figure legend) produced an output which is qualitatively as well as quantitatively indistinguishable from the minimal model with lower C3bBb half-life. This was true even when properdin was included in the model but was only allowed to bind C3bBb (dashed magenta line; $k_{13} = k_{23} = k_{24} = 0$), thereby generating C3bBbP with its long half-life. The blue, cyan and magenta curves are indistinguishable from each other as almost no quantitative difference is made by allowing increased C3bBb half-life either with or without properdin.

Properdin binding to C3bB alone (both closed and open forms together) (green dotted line; $k_{13} = k_{17} = 0$) had minimal impact on moving the solution trajectories towards the full properdin model. The most pronounced observation was when properdin was allowed to bind C3b (red solid line; $k_{17} = k_{23} = k_{24} = 0$), which predicted qualitatively similar C3 consumption and total C3 convertase (C3bBb+C3bBbP) response. This suggested that protection of C3b from FH/FI-mediated inactivation is crucial for positive regulation by properdin. This result is aligned with the observation from the minimal model which showed that the negative regulation also acts primarily through inactivation of C3b. However, it should be noted that protection of C3b from inactivation still did not explain the full impact of properdin and the interactions with C3bB and C3bBb appear to function synergistically.

Several authors have suggested that the main mechanism by which properdin positively regulates AP is by prolonging the half-life of the AP C3 convertase C3bBb (Fearon 1975; Hourcade 2006; Harboe and Mollnes 2008; Blatt et al. 2016). It has also been suggested that binding of properdin to C3b effectively prevents FH from binding and makes it available for FB to bind (Kouser et al. 2013). The quantitative analysis presented here has shown that prolonging the C3bBb half-life alone cannot reproduce the positive regulation by properdin. On the other hand, interactions with C3b as well as C3bB are necessary to positively regulate the AP. The current understanding that properdins main function is the stabilization of the C3 convertase is challenged by these results and should be examined in vitro to validate these observations.

We note that the observations from model simulations are dependent on parameter values and the structure of the underlying models. Sensitivity analyses were performed (supplementary material) for both the models to understand how each parameter influences the model output. We found that the outcome of the minimal model is most sensitive to synthesis and degradation rates of FH as well as binding/unbinding rate of FH with C3b, whereas the properdin model is additionally sensitive to synthesis and degradation rates of properdin. It is useful to note that neither model shows high sensitivity to the only parameter for which experimental measurement was not available ($k_{7}$, which is the Vmax for FD-mediated enzymatic cleavage of open C3bB).

We have focused on a subset of AP reactions, namely the fluid-phase reactions. However, complement pathology often manifests in a localized manner (e.g. AMD) where cell-surface-based regulation may have a bigger role to play. Although fluid-phase regulation suggests that the chief mechanism of properdin action is protection of C3b from inactivation, it is not clear whether the same conclusion will apply to surface-based complement activation in the presence of surface regulators. This will be governed by mechanisms and strength of negative surface regulators. The AP is a complex biochemical system involving fluid-phase as well as surface reactions and local as well as systemic manifestations. Part of this complexity was captured in a previous model by Zewde et al. (2016) and Zewde and Morikis (2018)). These models used mass-action kinetics for both surface-based and fluid-phase reactions. Surface-based CS reactions can be highly local and spatially inhomogeneous, which suggests that the assumptions required for mass-action kinetics to hold may be violated. Modelling approaches, which allow for spatial heterogeneity as well as description of cells as individual agents, may be needed to accurately predict local CS effects in its true complexity. Furthermore, quantitative as well as qualitative model predictions may be affected by the parameter uncertainty involved in large-scale models.

## Conclusion

With our parsimonious modelling approach we gained insight into the minimal machinery necessary for achieving physiological steady state in the AP. We have understood the essential role of negative regulation in stabilizing the system and controlling autonomous activation in the absence of a trigger.

Disruption of negative AP regulation results in pathological dysfunction. In C3 glomerulopathy caused by defective FH, for example, C3 is depleted systemically through uncontrolled AP activation. The positive regulation, on the other hand, raises the baseline of C3 convertase and may lower activation threshold.

In addition, we have demonstrated that FH-mediated negative regulation is driven by FI-mediated inactivation of C3b, thereby preventing the formation of the pro-convertase, while properdin counters this regulation primarily through protection of C3b from inactivation. This is in contrast to the long-held view that properdin exerts its positive regulation by prolonging the half-life of C3bBb and warrants further experimentation.

The AP displays a dichotomous response; it can exist in homoeostasis in the absence of an immune trigger and it can be activated in response to a trigger. The activation response is acute and can lead to complete depletion of C3 as informed by clinical observations (e.g. C3 glomerulopathy). Although the truncated model simulates the depletion of C3 as observed clinically in a few cases of C3 glomerulopathy, in chronic CS diseases the physiological response is likely to be less severe. Therefore, we hypothesize that the minimal model, with parameters in a “diseased” state (for example, reduced FH binding) is the better to simulate such conditions. Even though larger and more complex models of AP have recently been published in the literature, our simplified approach and emphasis on the use of experimentally available parameter values has provided biological insights into the regulation of AP.

### Electronic supplementary material

Below is the link to the electronic supplementary material.

Supplementary material 1 (docx 94 KB)

## Figures and Tables

**Fig. 8:**
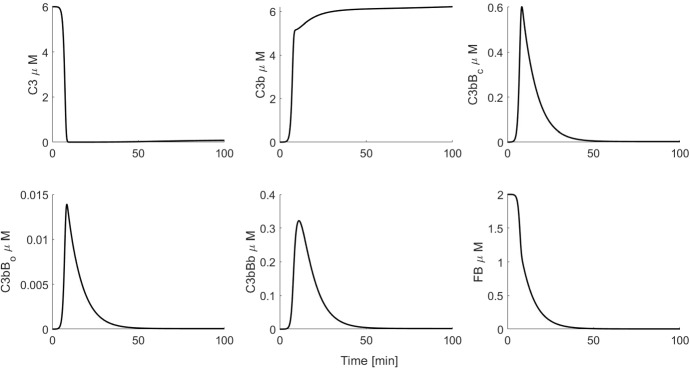
Simulation of the truncated minimal model of AP shows a strong activation response with C3 depletion and large C3bBb peak response. Initial conditions used were C3 $$= 6$$, FB $$ = 2$$ and zero for all other variables

**Fig. 9:**
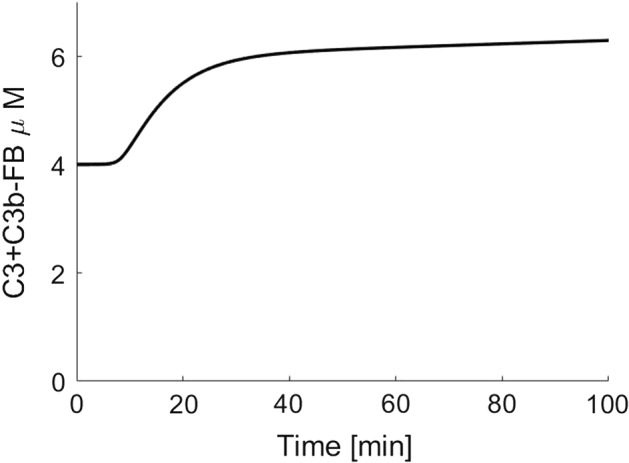
Simulation showing combined evolution of (C3 $+$ C3b − FB). Initial conditions used were C3 $= 6$, FB $= 2$ and zero for all other variables. It can be seen that there are three phases—an initial sta tionary phase, followed by a growth phase, which evolves into a slower growth phase at large times

**Fig. 10:**
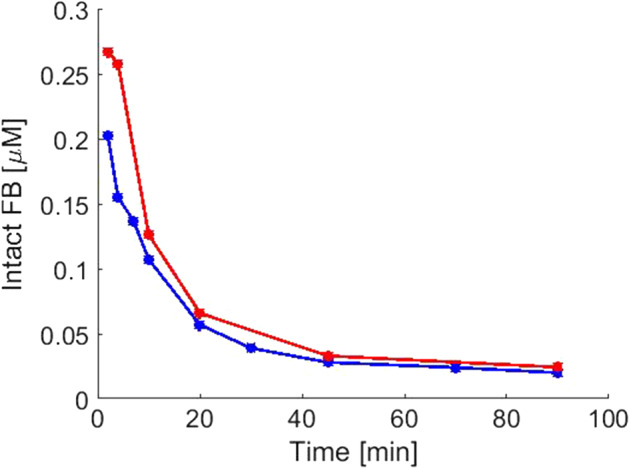
Levels of intact FB observed from two interactions (red and blue) of the experiment with C3b, FB and FD (Color figure online)

## Minimal Model

Figure 2 shows schematically the reactions in the minimal model. The respective model equations are

A.1 $$\begin{aligned} \dfrac{\mathrm{d}C3}{\mathrm{d}t}&= k_{s1} - d_{1}C3 -k_{1}C3 - \dfrac{k_{2}\cdot C3bBb \cdot C3}{k_{3} + C3},\nonumber \\ \dfrac{\mathrm{d}C3b}{\mathrm{d}t}&= k_{1}C3 + \dfrac{k_{2}\cdot C3bBb \cdot C3}{k_{3} + C3} - k_{4}C3b \cdot FB + k_{5}C3bB_c + k_{6}C3bBb\nonumber \\&\quad -k_{15}C3b\cdot FH + k_{16}C3bH + k_{21}C3bBbH,\nonumber \\ \dfrac{\mathrm{d}C3bB_c}{\mathrm{d}t}&= k_{4}C3b\cdot FB - k_{5}C3bB_c - k_{9}C3bB_c + k_{10}C3bB_o,\nonumber \\ \dfrac{\mathrm{d}C3bB_o}{\mathrm{d}t}&= k_{9}C3bB_c - k_{10}C3bB_o - \dfrac{k_{7}FD\cdot C3bB_o}{k_{8} + C3bB_o},\nonumber \\ \dfrac{\mathrm{d}C3bBb}{\mathrm{d}t}&= \dfrac{k_{7}FD\cdot C3bB_o}{k_{8} + C3bB_o} - k_{6}C3bBb - k_{25}C3bBb\cdot FH + k_{16}C3bBbH,\nonumber \\ \dfrac{\mathrm{d}FB}{\mathrm{d}t}&= k_{s2} - d_{2}FB - k_{4}C3b\cdot FB + k_{5}C3bB_c,\nonumber \\ \dfrac{\mathrm{d}FH}{\mathrm{d}t}&= k_{s3} - d_{3}FH -k_{15}C3b\cdot FH + k_{16}C3bH\nonumber \\&\quad - k_{25}C3bBb\cdot FH + k_{16}C3bBbH + \dfrac{k_{19}C3bH\cdot FI}{k_{20}+C3bH} + k_{21}C3bBbH, \nonumber \\ \dfrac{\mathrm{d}C3bH}{\mathrm{d}t}&= k_{15}C3b\cdot FH - k_{16}C3bH - \dfrac{k_{19}C3bH\cdot FI}{k_{20}+C3bH},\nonumber \\ \dfrac{\mathrm{d}C3bBbH}{\mathrm{d}t}&= k_{25}C3bBb\cdot FH - k_{16}C3bBbH - k_{21}C3bBbH. \end{aligned}$$

We use physiologically realistic concentrations of C3 $= 6\,\upmu \hbox {M}$, FB $= 2\,\upmu \hbox {M}$ and FH $= 3\,\upmu \hbox {M}$ as initial conditions (Alper and Rosen 1984; Scholl et al. 2008). (Corresponding ranges of physiologically realistic concentrations are given in Table 1.) Initial concentrations of all other intermediates are set to zero since no literature data are available about their steady-state levels.

### Steady States

Obtaining closed-form steady-state solution for Eq. (A.1) is not possible due to the complexity of the system. Therefore, we used simulation results to inform about different concentration scales at steady state and used this information to derive analytical solution of a reduced version of the minimal model.

Based on simulations, we observe that at steady-state species *C*3,  *FB* and *FH* are at micromolar concentrations, whereas all other species are at picomolar or lower concentrations. Thus, *C*3*b*, $C3bB_c$, $C3bB_o$, *C*3*bBb*, *C*3*bH* and *C*3*bBbH* are all $O(\varepsilon ) = 1e-6$ or lower at steady state.

This allows us to replace the Michaelis–Menten terms for FD-mediated cleaving of $C3bB_o$ and FI-mediated inactivation of *C*3*bH* by linear terms, since the respective $K_M$s are large, i.e. $k_{8},k_{20} \gg O(\varepsilon )$.

Further, we observe that certain parameter values are $O(10^1)$ or greater, whereas others are $O(10^0)$ or lower. For example, parameters such as $k_2,~k_{15},~k_{16},~k_{19}FI/k_{20}$ and $k_7 FD/k_8$ are all of $O(10^1)$ or greater. This allows us to choose the terms that would have greatest impact in a given ODE.

Using above observations, we arrive at the following reduced system of algebraic equations at steady state,

A.2 $$\begin{aligned} \dfrac{\mathrm{d}C3}{\mathrm{d}t}&= k_{s1} - d_{1}C3 -k_{1}C3 + O(\varepsilon ) = 0,\nonumber \\ \dfrac{\mathrm{d}C3b}{\mathrm{d}t}&= k_{1}C3 - k_{15}C3b\cdot FH + k_{16}C3bH + O(\varepsilon ) = 0,\nonumber \\ \dfrac{\mathrm{d}C3bB_c}{\mathrm{d}t}&= k_{4}C3b\cdot FB - k_{5}C3bB_c - k_{9}C3bB_c + k_{10}C3bB_o = 0,\nonumber \\ \dfrac{\mathrm{d}C3bB_o}{\mathrm{d}t}&= k_{9}C3bB_c - k_{10}C3bB_o - \dfrac{k_{7}FD}{k_{8} }C3bB_o = 0,\nonumber \\ \dfrac{\mathrm{d}C3bBb}{\mathrm{d}t}&= \dfrac{k_{7}FD}{k_{8} }C3bB_o - k_{6}C3bBb - k_{25}C3bBb\cdot FH + k_{16}C3bBbH = 0,\nonumber \\ \dfrac{\mathrm{d}FB}{\mathrm{d}t}&= k_{s2} - d_{2}FB + O(\varepsilon ) = 0,\nonumber \\ \dfrac{\mathrm{d}FH}{\mathrm{d}t}&= k_{s3} - d_{3}FH + O(\varepsilon ) = 0 \nonumber \\ \dfrac{\mathrm{d}C3bH}{\mathrm{d}t}&= k_{15}C3b\cdot FH - k_{16}C3bH - \dfrac{k_{19}FI}{k_{20}}C3bH = 0,\nonumber \\ \dfrac{\mathrm{d}C3bBbH}{\mathrm{d}t}&= k_{25}C3bBb\cdot FH - k_{16}C3bBbH - k_{21}C3bBbH = 0. \end{aligned}$$

The above system can be solved analytically, and the solution is

A.3 $$\begin{aligned} C3^*&= \dfrac{k_{s1}}{d_{1}+k_{1}},~~FB^* = \dfrac{k_{s2}}{d_{2}},~~FH^* = \dfrac{k_{s3}}{d_{3}},\nonumber \\ C3bH^*&= k_1 C3^* \dfrac{k_{20}}{k_{19}FI}, \nonumber \\ C3b^*&= \dfrac{C3bH^*}{FH^*}\left( k_{16} + \dfrac{k_{19}FI}{k_{20}}\right) \cdot \dfrac{1}{k_{15}} ,\nonumber \\ C3bB_c^*&= C3b^* FB^*\cdot \left( \dfrac{k_4}{(k_5 + k_9) + \dfrac{k_9 k_{10}}{k_{10} + k_7 FD/k_8}} \right) ,\nonumber \\ C3bB_o^*&= \dfrac{k_9}{k_{10} + k_{7}FD/k_8}\cdot C3bB_c^* ,\nonumber \\ C3bBb^*&= \dfrac{ k_{7}FD/k_8}{k_6+\left( \dfrac{k_{25}k_{21}FH^*}{k_{16}+k_{21}}\right) } C3bB_o^*,\nonumber \\ C3bBbH^*&= FH^*C3bBb^*\dfrac{k_{25}}{k_{16}+k_{21}}\nonumber \\ \end{aligned}$$

where asterisk denotes steady-state solutions. Numerical values of these steady states are presented in Table 3 in the “Appendix”, together with steady-state values obtained from simulations. It can be seen that the steady-state values from the reduced model compare well with the simulated values from the full minimal model.

**Table 3:** Steady-state levels of various species found using simulation of minimal and properdin models

Variable	Minimal model (analytical) ($\upmu \hbox {M}$)	Minimal model (simulation) ($\upmu \hbox {M}$)	Properdin model (simulation) ($\upmu \hbox {M}$)
*C*3	6.42	6.42	2.67
$C3b + C3bP$	1.7e−6	1.7e−6	7.4e−6
$C3bB_c + C3bB_cP$	4.7e−7	4.7e−7	3e−6
$C3bB_o + C3bB_oP$	1.06e−8	1.06e−8	2.15e−7
$C3bBb + C3bBbP$	3.42e−8	3.4e−8	5.5e−5
*FB*	2.39	2.39	2.39
*FH*	3.045	3.045	3.045
*C*3*bH*	5.15e−6	5.15e−6	1.7e−5
*C*3*bBbH*	1.66e−7	1.65e−7	1.9e−6
*P*	–	–	0.44

Steady-state levels from the minimal model are also determined mathematically after reducing the model based on different concentration and time scales

### Nondimensionalization

We use the following relationships for making the minimal model dimensionless.

A.4 $$\begin{aligned} \tau&= k_{16}t;~~x_{1} = C3/k_3;~~x_{6} = FB/k_3;~~x_{7} = FH/k_3; \nonumber \\ x_{2}&= C3b/e6;~~x_{3} = C3bB_c/e7;~~x_{4} = C3bB_o/e9;~~x_{5} = C3bBb/e8; \nonumber \\ x_{8}&= C3bH/e6;~~x_{9} = C3bBbH/e8, \end{aligned}$$

where $e6 = 1e-6$, $e7 = 1e-7$, $e8 = 1e-8$ and $e9 = 1e-9$. The resulting dimensionless system is given by the following equations

A.5 $$\begin{aligned} \dfrac{\mathrm{d}x_1}{d\tau }&= \varepsilon \left( \alpha _{1} - \alpha _{2} x_1 - \alpha _{3} x_1 - \alpha _{4}\dfrac{x_1 x_5}{1+x_1}\right) ,\nonumber \\ \dfrac{\mathrm{d}x_2}{d\tau }&= \alpha _5 x_1 + \alpha _{6}\sqrt{\varepsilon }\dfrac{x_1 x_5}{1+x_1} - \alpha _{7}\sqrt{\varepsilon } x_2 x_6 + \alpha _{8}\sqrt{\varepsilon } x_3 - \alpha _{10}x_2 x_7 + x_8 + \alpha _{11}\varepsilon x_9\nonumber \\ \dfrac{\mathrm{d}x_3}{d\tau }&= \alpha _{12}x_2 x_6 - \alpha _{13}\sqrt{\varepsilon } x_3 - \alpha _{14}\sqrt{\varepsilon } x_3 + \alpha _{15}\varepsilon x_4,\nonumber \\ \dfrac{\mathrm{d}x_4}{d\tau }&= \alpha _{16}x_3 - \alpha _{17}\sqrt{\varepsilon } x_4 - \alpha _{18}\sqrt{\varepsilon } x_4,\nonumber \\ \dfrac{\mathrm{d}x_5}{d\tau }&= \alpha _{19}\sqrt{\varepsilon } x_4 - \alpha _{20}\sqrt{\varepsilon } x_5 - \alpha _{21}x_5 x_7 + x_9,\nonumber \\ \dfrac{\mathrm{d}x_6}{d\tau }&= \varepsilon \left( \alpha _{22} - \alpha _{23} x_6 - \alpha _{24}\sqrt{\varepsilon } x_2 x_6 + \alpha _{25}\sqrt{\varepsilon } x_3\right) ,\nonumber \\ \dfrac{\mathrm{d}x_7}{d\tau }&= \varepsilon \left( \alpha _{26} - \alpha _{27} x_7 - \alpha _{28} x_2 x_7 + \alpha _{29} x_8 - \alpha _{30}\sqrt{\varepsilon } x_5 x_7 + \alpha _{31}\sqrt{\varepsilon } x9 + \alpha _{32}\varepsilon x_9\right) \nonumber \\ \dfrac{\mathrm{d}x_8}{d\tau }&= \alpha _{10}x_2 x_7 - x_8 - \alpha _{33}x_8,\nonumber \\ \dfrac{\mathrm{d}x_9}{d\tau }&= \alpha _{21}x_5 x_7 - x_9 - \alpha _{34}\sqrt{\varepsilon }x_9. \end{aligned}$$

where the parameters $\alpha _{n}$ are all *O*(1) and $\varepsilon = 1e-6$. The parameter groups, their names (a combination of $\alpha _n$ and $\varepsilon$ and values are listed in Table 4.

The following observations can be made by looking at the dimensionless model (Eq. A.5).

- Pathway precursors—such as C3, FB and FH ($x_1,~x_6$ and $x_7$) change very slowly as compared to pathway intermediates (all other species). This observation could be used for model reduction using singular perturbation analysis. However, that was not the focus of this work.
- FH ($$x_7)$$ binding to C3b ($$x_2$$) and to C3bBb $$(x_5)$$ occurs on the same time scale. However, the resulting C3bH ($$x_8$$) is more rapidly eliminated than C3bBbH ($$x_9$$). This may be responsible for the observation that FH exerts its negative regulation at C3b, rather than C3bBb.
- Synthesis and degradation reactions are very slow and are unlikely to significantly alter the dynamic response. However, they are crucial to obtain the correct steady-state response (as can be seen by the steady-state analysis).

**Table 4:** Table showing list of dimensionless parameter groups in the minimal model, their names and their numerical values

Parameter group	Name	Value	Parameter group	Name	Value
$k_{s1}/(k_3k_{16})$	$\alpha _{1}\varepsilon$	2.7e−6	$k_{7} FD/k_8 k_{16}$	$\alpha _{18}\sqrt{\varepsilon }$	0.074
$d_{1}/k_{16}$	$\alpha _{2}\varepsilon$	2e−6	$k_{7} FD e9/k_8 k_{16} e8$	$\alpha _{19}\sqrt{\varepsilon }$	0.0074
$k_{1}/k_{16}$	$\alpha _{3}\varepsilon$	5e−7	$k_{6}/k_{16}$	$\alpha _{20}\sqrt{\varepsilon }$	0.0024
$k_{2}e8/k_3k_{16}$	$\alpha _{1}\varepsilon \sqrt{\varepsilon }$	9.3e−10	$k_{25} k_3/k_{16}$	$\alpha _{21}$	0.273
$k_1 k_3/k_{16}e6$	$\alpha _{5}$	3.005	$k_{s2}/k_3 k_{16}$	$\alpha _{22}\varepsilon$	6.9e−7
$k_2 e8/k_{16} e6$	$\alpha _{6}\sqrt{\varepsilon }$	0.0055	$d_{2}/k_{16}$	$\alpha _{23}\varepsilon$	1.7e−6
$k_4 k_3/k_{16}$	$\alpha _{7}\sqrt{\varepsilon }$	0.0245	$k_{4} e6/k_{16}$	$\alpha _{24}\varepsilon \sqrt{\varepsilon }$	4e−9
$k_5 e7/k_{16} e6$	$\alpha _{8}\sqrt{\varepsilon }$	0.0035	$k_{5} e7/k_{16} k_3$	$\alpha _{25}\varepsilon \sqrt{\varepsilon }$	6e−10
$k_6 e8/k_{16} e6$	$\alpha _{9}\varepsilon$	2.36e−5	$k_{s3}/k_3 k_{16}$	$\alpha _{26}\varepsilon$	8e−7
$k_{15}/k_{3} k_{16}$	$\alpha _{10}$	0.273	$d_{3}/k_{16}$	$\alpha _{27}\varepsilon$	1.2e−6
$k_{21} e8/k_{16} e6$	$\alpha _{11}\varepsilon$	4.2e−5	$k_{15} e6/k_{16}$	$\alpha _{28}\varepsilon$	1.6e−6
$k_3 k_4 e6/k_{16} e7$	$\alpha _{12}$	0.245	$e6/k_{3}$	$\alpha _{29}\varepsilon$	1.7e−7
$k_{5}/k_{16}$	$\alpha _{13}\sqrt{\varepsilon }$	0.035	$k_{25} e8/k_{16}$	$\alpha _{30}\varepsilon \sqrt{\varepsilon }$	1.6e−8
$k_{9}/k_{16}$	$\alpha _{14}\sqrt{\varepsilon }$	0.0017	$e8/k_{3}$	$\alpha _{31}\varepsilon \sqrt{\varepsilon }$	1.7e−9
$k_{10} e9/k_{16} e7$	$\alpha _{15}\varepsilon$	5.48e−6	$k_{21}e8/k_{16}k_3$	$\alpha _{32}\varepsilon ^2$	7.3e−12
$k_{9} e7/k_{16} e9$	$\alpha _{16}$	0.17	$k_{19}FI/k_{16}k_{20}$	$\alpha _{33}$	0.64
$k_{10}/k_{16}$	$\alpha _{17}\sqrt{\varepsilon }$	5.48e−4	$k_{21}/k_{16}$	$\alpha _{34}\sqrt{\varepsilon }$	0.0043

## Truncated Minimal Model

Mutations in FH or antibodies against FH can lead to quantitative or functional depletion of FH. We simulate this depletion by setting FH synthesis rate $k_{s3} = 0$ and using $FH(t=0) = 0$ as the initial condition. This results in the truncated minimal model. The model equations in the absence of FH are

B.1 $$\begin{aligned} \dfrac{\mathrm{d}C3}{\mathrm{d}t}&= k_{s1} - d_{1}C3 -k_{1}C3 - \dfrac{k_{2}\cdot C3bBb\cdot C3}{k_{3} + C3},\nonumber \\ \dfrac{\mathrm{d}C3b}{\mathrm{d}t}&= k_{1}C3 + \dfrac{k_{2}\cdot C3bBb\cdot C3}{k_{3} + C3} - k_{4}C3b\cdot FB + k_{5}C3bB_c + k_{6}C3bBb,\nonumber \\ \dfrac{\mathrm{d}C3bB_c}{\mathrm{d}t}&= k_{4}C3b\cdot FB - k_{5}C3bB_c - k_{9}C3bB_c + k_{10}C3bB_o,\nonumber \\ \dfrac{\mathrm{d}C3bB_o}{\mathrm{d}t}&= k_{9}C3bB_c - k_{10}C3bB_o - \dfrac{k_{7}FD\cdot C3bB_o}{k_{8} + C3bB_o},\nonumber \\ \dfrac{\mathrm{d}C3bBb}{\mathrm{d}t}&= \dfrac{k_{7}FD\cdot C3bB_o}{k_{8} + C3bB_o} - k_{6}C3bBb,\nonumber \\ \dfrac{\mathrm{d}FB}{\mathrm{d}t}&= k_{s2} - d_{2}FB- k_{4}C3b\cdot FB + k_{5}C3bB_c. \end{aligned}$$

Simulation of this model shows greatly different behaviour as compared to the minimal model. It shows a strong activation response with near-complete depletion of C3 and FB after a delay and a corresponding rise in concentrations of all the intermediates (Fig. 8).

The qualitative behaviour of the activation response of the minimal model matches with the simulations of Korotaevskiy and co-workers (Korotaevskiy et al. 2009). An experiment by Pangburn and co-workers with purified C3, FB and FD showed complete consumption of C3 within 2 minutes (Pangburn et al. 1981). This complete and quick C3 depletion matches with our simulations. (The subsequent small rise in C3 levels is attributable to *de novo* synthesis). A similar experiment with purified C3b, FB and FD (at a low concentration) was conducted at GSK. The levels of intact FB were monitored using ELISA at various time points (experimental methods and data in “Appendix D”). Intact FB was almost entirely depleted within 90 minutes (Fig. 10).

Additionally, it has been shown that serum depleted in functional negative regulators of AP displays maximal AP activation (Pangburn and Rawal 2002). We note that the AP activation observed in the model is also autonomous, i.e. it occurs in the absence of an external trigger. This observation is aligned with experiments with purified AP components (Pangburn et al. 1981) and serum depleted in negative regulators (Pangburn and Rawal 2002). Thus, truncated model together with minimal model shows the strong negative regulation of AP by FH in line with experimental data.

### Nondimensionalization

Truncated model simulation reveals that certain reactions occur on a faster time scale than others. In this section, we discuss the nondimensionalization of the truncated model and the insights gained from it. We use the following scaling factors, inspired by the simulated values of all species in the truncated model.

B.2 $$\begin{aligned} \tau&= k_{2}t;~~x_{1} = C3/e0;~~x_{2} = C3b/e0;~~x_{6} = FB/e0; \nonumber \\ x_{3}&= C3bB_c/e1;~~x_{4} = C3bB_o/e3;~~x_{5} = C3bBb/e1; \end{aligned}$$

where $e0 = 1e0 = 1$, $e1 = 1e-1$ and $e3 = 1e-3$. Further, we observe that maximum $C3bB_{o}$ concentration reached is small as compared to $k_{8}$, which allows us to replace the Michaelis–Menten term with a linear term. Thus, $k_7 FD C3bB_o/(k_8 + C3bB_o) \approx k_7 FD C3bB_o/k_8$. The resulting dimensionless system is

B.3 $$\begin{aligned} \dfrac{\mathrm{d}x_1}{d\tau }&= \beta _1 \varepsilon _1^2 - \beta _2 \varepsilon _1^2 x_1 - \beta _3 \varepsilon _1^2 x_1 - \beta _4 x_5\dfrac{x_1}{\beta _5 + x_1},\nonumber \\ \dfrac{\mathrm{d}x_2}{d\tau }&= \beta _3 \varepsilon _1^2 x_1 + \beta _4 x_5\dfrac{x_1}{\beta _5 + x_1} - \beta _6 \varepsilon _1 x_2 x_6 + \beta _7 \varepsilon _1 x_3 + \beta _8 \varepsilon _1 x_5,\nonumber \\ \dfrac{\mathrm{d}x_3}{d\tau }&= \beta _9 \sqrt{\varepsilon _1} x_2 x_6 - \beta _{10} \sqrt{\varepsilon _1} x_3 - \beta _{11} \varepsilon _1 x_3 + \beta _{12}\varepsilon _1^2 x_4,\nonumber \\ \dfrac{\mathrm{d}x_4}{d\tau }&= \beta _{13} x_3 - \beta _{14}\varepsilon _1 x_4 - \beta _{15} x_4,\nonumber \\ \dfrac{\mathrm{d}x_5}{d\tau }&= \beta _{16}\varepsilon _1 x_4 - \beta _{17}\varepsilon _1 x_5,\nonumber \\ \dfrac{\mathrm{d}x_6}{d\tau }&= \beta _{18}\varepsilon _1^2 - \beta _{19}\varepsilon _1^2 x_2 - \beta _6 \varepsilon _1 x_2 x_6 + \beta _7 \varepsilon _1 x_3, \end{aligned}$$

where $\beta _n$ are all *O*(1) parameters and $\varepsilon _1 = 1e-3$. The parameter groups and their numerical values are listed in Table 5.

**Table 5:** Table showing list of dimensionless parameter groups in the truncated model, their names and their numerical values

Parameter group	Name	Value	Parameter group	Name	Value
$k_{s1}/(e0 k_{2})$	$\beta _{1}\varepsilon _1^2$	2.9e−5	$k_{9}/k_2$	$\beta _{11}\varepsilon _1$	3.1e−3
$d_{1}/k_{2}$	$\beta _{2}\varepsilon _1^2$	3.6e−6	$k_{10} e3/k_2 e1$	$\beta _{12}\varepsilon _1^2$	9.9e−6
$k_{1}/k_{16}$	$\beta _{3}\varepsilon _1$	9.3e−7	$k_{9}e1/k_2 e3$	$\beta _{13}$	0.308
*e*1/*e*0	$\beta _{4}$	0.1	$k_{10}/k_{2}$	$\beta _{14}\varepsilon _1$	9.9e−4
$k_3/e0$	$\beta _{5}$	5.86	$k_{7}FD/k_8 k_{2}$	$\beta _{15}$	0.135
$k_{4}e0/k_2$	$\beta _{6}\varepsilon _1$	7.6e−3	$k_{7}FD e3/k_8 k_{2} e1$	$\beta _{16}\varepsilon _1$	1.3e−3
$k_5 e1/k_2$	$\beta _{7}\varepsilon _1$	6.4e−3	$k_{6}/k_{2}$	$\beta _{17}\varepsilon _1$	4.3e−3
$k_6 e1/k_2 e0$	$\beta _{8}\varepsilon _1$	4.2e−4	$k_{s2}/k_2 e0$	$\beta _{18}\varepsilon _1^2$	7.5e−6
$k_4 e0^2/k_{2} e1$	$\beta _{9}\sqrt{\varepsilon _1}$	0.076	$d_{2}/k_2$	$\beta _{19}\varepsilon _1^2$	3.1e−6
$k_{5}/k_2$	$\beta _{10}\sqrt{\varepsilon _1}$	0.065			

Based on the dimensionless truncated model and Fig. 8, we make the following observations.

- Synthesis and degradation of C3 ($x_1$) and FB ($x_6$) is small as compared to the rest of the reactions.
- The rapid decline in C3 ($x_1$) is caused by C3bBb ($x_5$)-mediated cleavage of C3.
- The delay in the C3 ($x_1$) decline (or resultant C3b ($x_2$) rise) occurs due to the delay in accumulation of C3bBb ($x_5$).
- Conversion of $x_2$ to $x_5$ occurs through a cascade of $x_3$, $x_4$, and $x_5$. Thus, the total delay in C3 decline can be given as the sum of delays throughout the cascade.
- Observing the rate of change of (C3 $+$ C3b − FB) (i.e. $(x_1+x_2-x_6)$) is informative. In Fig. 9, we plot the combined evolution of (C3 $+$ C3b − FB).
  $$\begin{aligned} \dfrac{\mathrm{d}(x_1+x_2-x_6)}{\mathrm{d}t} = \beta _8 \varepsilon _1 x_5 + O(\varepsilon _1^2). \end{aligned}$$
  Thus, at early time, $d(x_1+x_2-x_6)/dt = 0$, and this quantity remains constant at 3.8 (from initial data). The subsequent rise on the longer time scale is driven initially by $x_5$. Synthesis and degradation reactions for C3 and FB provide a correction at very large time scales.

### Steady-State Analysis

Dimensionless version of the truncated model shows that synthesis and degradation reactions are $O(\varepsilon _1^2)$. When we ignore these terms, the truncated model turns into a closed system. Conservation laws can then be applied, and in the absence of synthesis/degradation reactions, we have,

$$\begin{aligned} C3 + C3b + C3bB_c + C3bB_o + C3bBb = Constant = C3(t=0), \end{aligned}$$

and

$$\begin{aligned} C3bB_c + C3bB_o + C3bBb + Bb + FB = Constant = FB(t=0), \end{aligned}$$

where the evolution of Bb is given as

$$\begin{aligned} \dfrac{\mathrm{d}Bb}{\mathrm{d}t} = k_6 C3bBb. \end{aligned}$$

Using these conservation laws, it can be shown that the steady state of the closed truncated system is

B.4 $$\begin{aligned} C3&= 0,~C3b = C3(t=0) = 6\,\upmu \hbox {M},\nonumber \\ C3bB_c&= 0,~ C3bB_o = 0,~ C3bBb = 0,\nonumber \\ FB&= 0. \end{aligned}$$

This result is in agreement with simulations in Fig. 8, except for the slow $O(\varepsilon _1^2)$ rise in C3 and C3b levels, which is attributable to the contribution from the synthesis/degradation reactions. The analytical steady state also matches the in vitro studies such as that reported in Fig. 10, and in Pangburn and Rawal (2002).

## Properdin Model

C.1 $$\begin{aligned} \dfrac{\mathrm{d}C3}{\mathrm{d}t}&= k_{s1} - d_{1}C3 -k_{1}C3 - \dfrac{k_{2}\cdot (C3bBb+C3bBbP)\cdot C3}{k_{3} + C3},\nonumber \\ \dfrac{\mathrm{d}C3b}{\mathrm{d}t}&= k_{1}C3 + \dfrac{k_{2}\cdot (C3bBb+C3bBbP)\cdot C3}{k_{3} + C3} - k_{4}C3b\cdot FB \nonumber \\&\quad + k_{5}C3bB_c + k_{6}C3bBb\nonumber \\&\quad -k_{15}C3b\cdot FH + k_{16}C3bH + k_{21}C3bBbH - k_{13}C3b\cdot P \nonumber \\&\quad + k_{14}C3bP + k_{22}C3bBbP,\nonumber \\ \dfrac{\mathrm{d}C3bB_c}{\mathrm{d}t}&= k_{4}C3b\cdot FB - k_{5}C3bB_c - k_{9}C3bB_c + k_{10}C3bB_o \nonumber \\&\quad - k_{23}C3bB_{c}\cdot P + k_{14}C3bB_{c}P,\nonumber \\ \dfrac{\mathrm{d}C3bB_o}{\mathrm{d}t}&= k_{9}C3bB_c - k_{10}C3bB_o \nonumber \\&\quad - \dfrac{k_{7}FD\cdot C3bB_o}{k_{8} + C3bB_o + C3bB_{o}P} - k_{24}C3bB_{o}\cdot P + k_{14}C3bB_{o}P,\nonumber \\ \dfrac{\mathrm{d}C3bBb}{\mathrm{d}t}&= \dfrac{k_{7}FD\cdot C3bB_o}{k_{8} + C3bB_o + C3bB_{o}P} - k_{6}C3bBb - k_{25}C3bBb\cdot FH \nonumber \\&\quad + k_{16}C3bBbH\nonumber \\&\quad - k_{17}C3bBb\cdot P + k_{18}C3bBbP,\nonumber \\ \dfrac{\mathrm{d}FB}{\mathrm{d}t}&= k_{s2} - d_{2}FB - k_{4}(C3b+C3bP)\cdot FB + k_{5}(C3bB_c + C3bB_{o}P),\nonumber \\ \dfrac{\mathrm{d}FH}{\mathrm{d}t}&= k_{s3} - d_{3}FH -k_{15}C3b\cdot FH + k_{16}C3bH\nonumber \\&\quad - k_{25}C3bBb\cdot FH + k_{16}C3bBbH \nonumber \\&\quad + \dfrac{k_{19}C3bH\cdot FI}{k_{20}+C3bH} + k_{21}C3bBbH, \nonumber \\ \dfrac{\mathrm{d}C3bH}{\mathrm{d}t}&= k_{15}C3b\cdot FH - k_{16}C3bH - \dfrac{k_{19}C3bH\cdot FI}{k_{20}+C3bH},\nonumber \\ \dfrac{\mathrm{d}C3bBbH}{\mathrm{d}t}&= k_{25}C3bBb\cdot FH - k_{16}C3bBbH - k_{21}C3bBbH,\nonumber \\ \dfrac{\mathrm{d}P}{\mathrm{d}t}&= k_{s4} - d_{4}P - k_{13}P\cdot C3b - k_{23}P\cdot C3bB_{c} - k_{24}P\cdot C3bB_{o} \nonumber \\&\quad + k_{14}(C3bP + C3bB_{c}P + C3bB_{o}P)\nonumber \\&\quad -k_{17}C3bBb\cdot P + k_{18}C3bBbP + k_{22}C3bBbP, \nonumber \\ \dfrac{\mathrm{d}C3bP}{\mathrm{d}t}&= k_{13}C3b\cdot P - k_{14}C3bP - k_{4}C3bP\cdot FB + k_{5}C3bB_{o}P, \nonumber \\ \dfrac{\mathrm{d}C3bB_{c}P}{\mathrm{d}t}&= k_{23}C3bB_{c}\cdot P - k_{14}C3bB_{c}P - k_{9}C3bB_{c}P + k_{10}C3bB_{o}P, \nonumber \\ \dfrac{\mathrm{d}C3bB_{o}P}{\mathrm{d}t}&= k_{24}C3bB_{o}\cdot P - k_{14}C3bB_{o}P + k_{9}C3bB_{c}P - k_{10}C3bB_{o}P \nonumber \\&\quad +k_{4}C3bP\cdot FB - k_{5}C3bB_{o}P - \dfrac{k_{7}FD\cdot C3bB_{o}P}{k_{8} + C3bB_o + C3bB_{o}P}, \nonumber \\ \dfrac{\mathrm{d}C3bBbP}{\mathrm{d}t}&= \dfrac{k_{7}FD\cdot C3bB_{o}P}{k_{8} + C3bB_o + C3bB_{o}P} + k_{17}C3bBb\cdot P \nonumber \\&\quad - k_{18}C3bBbP - k_{22}C3bBbP. \end{aligned}$$

We use physiologically realistic concentrations of $C3 = 6\,\upmu \hbox {M}$, $FB = 2\,\upmu \hbox {M}$, $FH = 3\,\upmu \hbox {M}$ and $P = 0.3\,\upmu \hbox {M}$ as initial conditions (Alper and Rosen 1984; Scholl et al. 2008). Initial concentrations of all other intermediates are set to zero since no literature data are available about their steady-state levels.

### Steady States

The properdin model (Eq. C.1) is not amenable to steady-state analysis owing to the increased complexity. However, the steady-state values can be obtained by simulating the model (Eq. C.1) with parameter values from Table 1. The steady-state values are listed in Table 3.

## Experimental Methods: FB Consumption Assay

### Protein Parameters

Mature complement C3b, FB and FD protein sequences were obtained from Uniprot.org and processed using SeqBuilder and Protean (DNASTAR Lasergene core suite 10). Cysteines were assumed in pair and as many hydrogen atoms as assumed paired cysteines were subtracted from the theoretical molar weights. Mass extinction coefficients were calculated as the ratio molar weight over molar extinction coefficient (Table 6).

### Protein Titration

Purified proteins (Complement technology Inc) were thawed on wet ice and centrifuged 5min, 17,000g, 4C to eliminate potential precipitates. Protein titre of the supernatants was determined by A280 method on Ultrospec 3300 pro (Amersham Biosciences).

### Enzyme Reaction

All proteins were prediluted in DPBS (Gibco # 14190) supplemented with 7mM magnesium chloride (1M solution, Sigma) and 0.05% Brij35 (30% solution, Sigma). Factor B and Factor D were premixed and brought to 37C in 1 well of a 96-well polystyrene plate in Thermo-Shaker PST-60HL-4 (Biosan). C3b was added extemporaneously in order to initiate Factor B conversion. Plate was sealed and incubated in motionless fashion. Final FB, FD and C3b concentrations were, respectively, of 0.0018 $\upmu$g/mL, 20 $\upmu$g/mL and 120 $\upmu$g/mL in 275 $\upmu$L final volume. $25\upmu \hbox {L}$ sample was taken at multiple timepoints ranging 2, 4, 7, 10, 20, 30, 45, 70 and 90min, and neutralized by 1:1 dilution in DPBS supplemented with 100mM EDTA, and 20 $\upmu$M proprietary inhibitor in new plate. Reaction was carried in triplicate.

**Table 6:** Determination of protein mass extinction coefficients

	FD	FB	C3b
Molar weight estimate (g/mol)	24,397	82,990	175,249
Molar extinction coefficient	28,960	123,545	178,075
Mass extinction coefficient (g/L per A280 unit)	0.8424	0.6717	0.9841

### Data

FB was quantified using a propitiatory sandwich ELISA (manuscript in preparation). Similar methods can be found in the literature (Crowther et al. 1990; Oppermann et al. 1990). The intact FB data thus obtained were converted into micromolar concentrations and are plotted in Fig. 10.
