# Supplementary material for: Mathematical Modelling of Alternative Pathway of Complement System
Source: Bull Math Biol. 2020 Feb 15;82(2):33. doi: 10.1007/s11538-020-00708-z (PMC7024062; doi:10.1007/s11538-020-00708-z)
Supplement: Supplementary file 1 — Supplementary material 1 (docx 94 KB) [file 11538_2020_708_MOESM1_ESM.docx]

**Supplementary material – Sensitivity analysis**

The sensitivity analysis was performed to determine the influence of various parameters. A parameter P was varied at a time and the change in steady state C3bBb values was recorded. The sensitivity index S was calculated using the formula

$$S=\frac{abs(C3bBb\left( Pj+\Delta Pj \right)-C3bBb\left( Pj \right))}{\Delta Pj}*\frac{Pj}{C3bBb(Pj)}$$

Where $Pj$ denotes the $jth$parameter, $\Delta Pj$ denotes the change in the $jth$parameter and $C3bBb(Pj)$ and $C3bBb(Pj+ \Delta Pj)$ denote the steady state values of C3bBb at the original and new parameter value respectively. The sensitivity index $S$ is normalized with parameter size as well as the original C3bBb steady state for comparability. Figures A and B show the relative sensitivity indices *S* for minimal and properdin models, respectively.


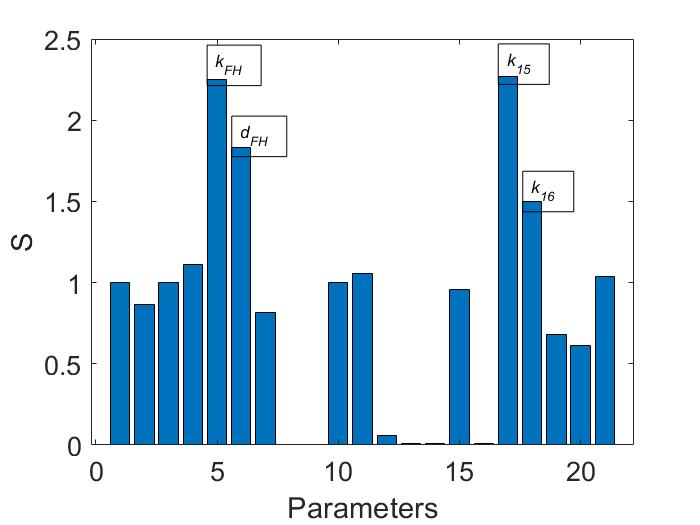


Figure A: Results of sensitivity analysis of the minimal model.


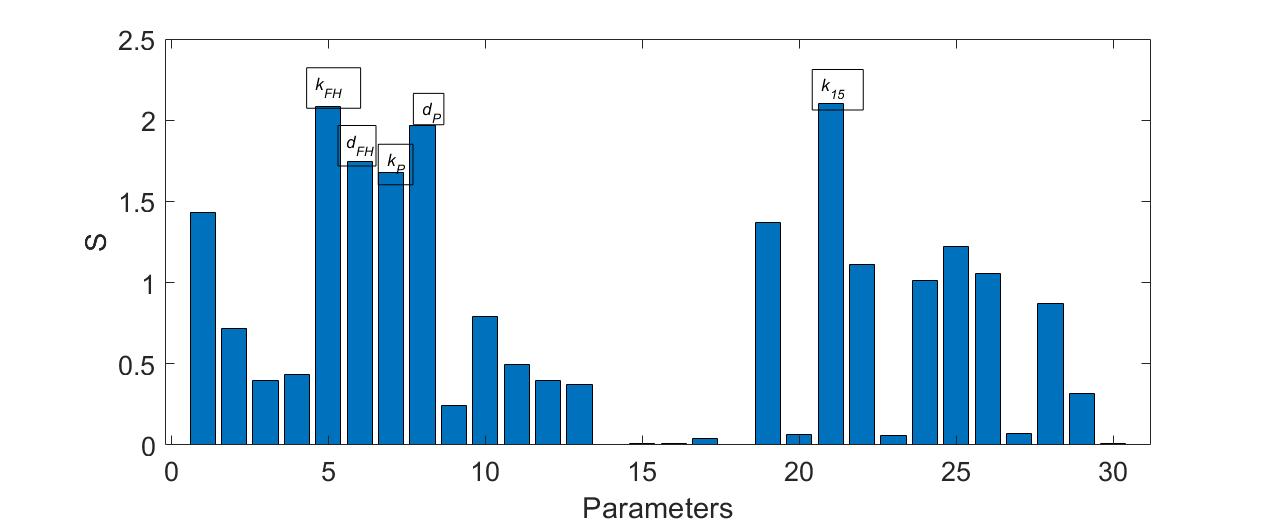


Figure B: Results of sensitivity analysis of the properdin model.
